# The outbreak of monkeypox: A clinical overview

**DOI:** 10.4102/safp.v64i1.5613

**Published:** 2022-11-03

**Authors:** Ramprakash Kaswa, Arun Nair, Klaus B. von Pressentin

**Affiliations:** 1 Department of Family Medicine and Rural Health, Faculty of Health sciences, Walter Sisulu University, Mthatha, Eastern Cape, South Africa; 2 Department of Family Medicine, Mthatha Regional Hospital, Eastern Cape, South Africa; 3 Department of Family Medicine, University of the Free State, Kimberley, South Africa; 4 Robert Mangaliso Sobukwe Hospital, Northern Cape Department of Health, Kimberley, South Africa; 5 Division of Family Medicine, School of Public Health and Family Medicine, Faculty of Health Sciences, University of Cape Town, Cape Town, South Africa

**Keywords:** Monkeypox, global health, smallpox, vaccine, zoonotic

## Abstract

The development of new zoonotic diseases such as coronavirus disease 2019 (COVID-19) and monkeypox that can cause epidemics and high mortality rates have significantly threatened global health security. However, the increasing number of people with no immunity to poxvirus because of the end of the smallpox vaccination programme has created a vulnerable population for the monkeypox outbreak. On 23 July 2022, it was announced that the World Health Organization’s director-general has determined that the multicountry outbreak of monkeypox constitutes a Public Health Emergency of International Concern. The monkeypox virus is an orthopoxvirus that causes a disease with symptoms similar to smallpox but less severe. Many unanswered questions remain regarding monkeypox’s pathogenesis, transmission and host reservoir. There is currently no evidence that transmission by individuals can sustain zoonotic infections during human-to-human transmissions; the continued emergence of these pathogens highlights the interconnectedness of animals and humans. The increasing number of monkeypox cases outside the endemic region has highlighted the need for effective global capacity building to prevent the spread of the disease and its impact on global health security. The priority now is to stop the spread of the disease and protect frontline healthcare workers and the most vulnerable individuals. This article aims to comprehensively analyse the various aspects of the transmission and epidemiology of monkeypox. It also explores possible diagnostic techniques, therapeutics and prevention strategies. A key recommendation is that primary care and public health professionals are expected to increase their efforts to be vigilant and contain any potential outbreaks.

## Background

From 01 January 2022 to 15 September 2022, there have been over 60 320 laboratory-confirmed cases of monkeypox and 23 deaths reported to the World Health Organization (WHO) by 103 member states.^1^ The majority of these cases were reported by the WHO European Region, followed by the Americas, Africa, the Eastern Mediterranean and the Western Pacific.^2^ To date, five laboratory-confirmed cases of monkeypox have been reported in South Africa.^2,3^ While a global map maintained by the Centers for Disease Control and Prevention (CDC) reported 61 282 confirmed cases across 74 locations, what was concerning was that 68 locations included countries that had not reported monkeypox previously.^4^ On 23 July 2022, following the second meeting of the International Health Regulations (2005) (IHR) Emergency Committee, it was announced that the WHO’s director-general had determined that the multicountry outbreak of monkeypox constituted a public health emergency of international concern.^5^

Monkeypox is a contagious zoonotic disease caused by the double-stranded DNA virus of the *Orthopoxvirus* genus in the Chordopoxvirinae subfamily and Poxviridae family.^6^ There are currently over 80 poxviruses known to science, and these have been isolated from various animals, mammals and birds.^7^ Two main strains of the human monkeypox virus, the Congo Basin clade and the West African clade, have been identified. The Congo Basin clade is more virulent and transmissible.^8^ The overall case-fatality rate for monkeypox ranges from 1% to 10%.^7^

The first known case of monkeypox was discovered in 1958 in Denmark. It was caused by a pox-like disease that affected a group of monkeys that were kept for research.^9^ The first human case of monkeypox was recorded in the Democratic Republic of Congo in 1970.^10^ It is believed that the virus can be introduced to humans through infected rodents. The monkeypox virus can be found in various animal species, such as tree squirrels, primates and rope squirrels in endemic areas. It is also believed that certain types of rodents are the carriers of the disease.^7,11^ Although monkeypox infections in humans are relatively rare, they can still occur in endemic areas.

The endemic countries for monkeypox are the Central African Republic, Cameroon, Ghana, Nigeria, Sierra Leone, Gabon, Ivory Coast, Liberia and the Democratic Republic of Congo.^9^ In the past, countries such as South Sudan and Benin have imported monkeypox. Frequently outbreaks of the West African clade are reported from Nigeria and Cameroon.^7^ All the endemic countries reported new disease cases as the multicountry outbreak has continued since January 2022. Usually, the cases of the monkeypox disease in other countries are linked to travel to endemic regions. However, the current outbreak is different from past travel-related outbreaks.^2,12^

The number of people with no immunity to smallpox increased after the vaccination programme ended, resulting in a vulnerable population.^13^ Although there is currently no evidence that person-to-person transmission can sustain the ongoing outbreaks of zoonotic infections in humans, the≈increasing number of people infected with these pathogens highlights the interconnectedness of humans, animals and the environment.^14,15^ The importance of implementing a ‘One Human–Environmental–Animal Health’ approach to controlling zoonotic infections needs to be acknowledged by policymakers.^15^ Despite progress, more work is needed to achieve a more effective and sustainable solution.

## Clinical presentation of monkeypox

The initial symptoms of monkeypox are usually fever, headache and fatigue.^16^ The incubation period for monkeypox is usually 7 to 14 days, but it can range from 5 to 21 days. During the prodromal phase, which lasts for about a week, patients may experience fever, back pain, headache, muscle aches and lymphadenopathy.^16,17^ After the fever subsides, the second phase usually begins with deep well-circumscribed vesicular, pustular and macular lesions. A centrifugal rash can start on the face and then spread to other body parts. It can also involve the mucous membranes of the oral cavity, genitalia, conjunctiva and cornea. The rash can progress through various other stages. It can then crust over and desquamate over 2 to 3 weeks.^8,18^

Some patients present with atypical symptoms of monkeypox, such as a few lesions localised to the perineal or genital area. They can also develop a rash before constitutional or prodromal symptoms such as fever and fatigue.^10^ Lymphadenopathy is a common feature of this illness and can usually appear as early as the first week of illness.^10,16^

**FIGURE 1:**
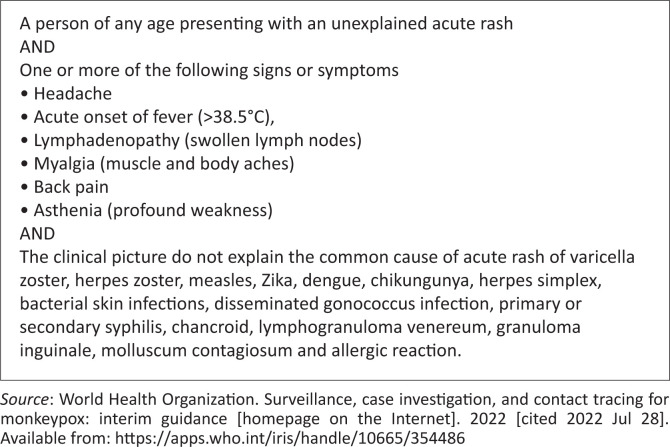
Suspected case definition of monkeypox.

**FIGURE 2:**
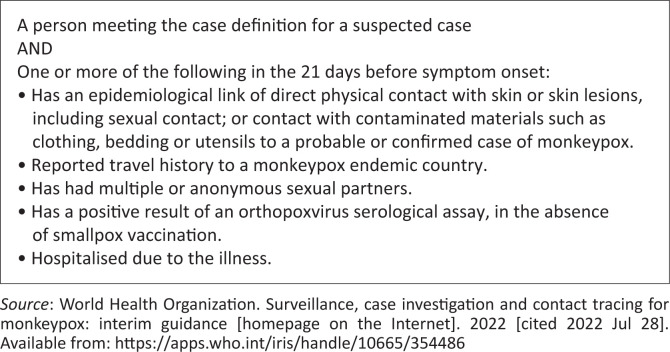
Probable case of monkeypox case definition.

Individuals who have not been vaccinated against smallpox and present in a nonendemic country with an acute rash on their hands and feet may be infected with the disease. However, the common causes of acute rash are viral infections, such as smallpox, chickenpox, rubella, syphilis, chikungunya and dengue.^6,11^ Figure 1 and Figure 2 demonstrate monkeypox’s suspected and probable case definition.

## Transmission of monkeypox

Humans can be infected with monkeypox through contact with mucocutaneous or infectious skin lesions.^19^ This can occur in various contact forms such as skin-to-skin, mouth-to-mouth or respiratory droplets. The virus can enter the body through broken skin, the respiratory tract or the oral and pharyngeal surfaces.^6,19,20^ In addition to skin lesions, monkeypox can also be transmitted from the environment to humans through the presence of infectious particles. These particles can then be inhaled and land on the broken skin or other mucosal membranes. The monkeypox virus can be further spread in endemic areas by handling infected bush meat through contact with bodily fluids and contaminated objects.^19^

**FIGURE 3:**
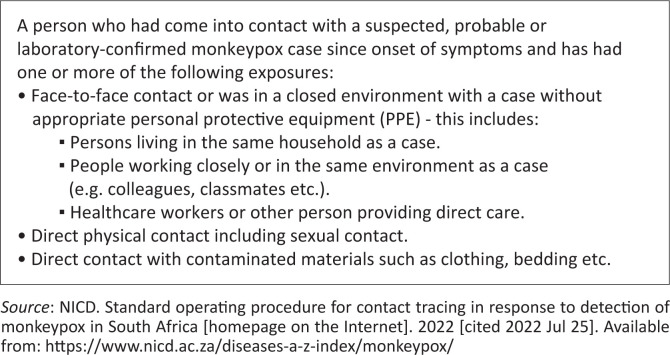
Contact definition of monkeypox.

While the initial symptoms of the disease can appear for up to two weeks, infected individuals can remain contagious.^2,19^ Although the exact period during which patients are infectious can vary, most will be considered contagious until their skin lesions have crusted and their scabs have disappeared.^11^ It is believed that prolonged exposure to the respiratory tract and proximity to infected individuals are the factors that can trigger the transmission of monkeypox. However, it is not yet clear how sexual transmission can also occur. During pregnancy, the virus can cross the placenta and cause intrauterine exposure to the foetus.^7,11^ In addition, individuals in contact with infected health workers and household members are at increased risk of getting the disease.^19^ Figure 3 demonstrates the contact definition of monkeypox.

## Isolation criteria for monkeypox exposure

Per the case definition criteria, travel to an endemic country within the past 21 days, before the appearance of symptoms, is an essential criterion for assessing the probability of monkeypox.^21^ However, people do not need to stay away from work after exposure to monkeypox. The WHO does not recommend quarantine or work restrictions for exposed individuals during the contact tracing. The WHO encourages all contacts to follow proper hand hygiene and respiratory etiquette.^19,21^ It also recommends avoiding physical contact with immunocompromised individuals, pregnant women, children and sexual abstinence. Nonessential travel is also discouraged.^21^

If asymptomatic contacts can still perform regular activities, such as school and work, they should refrain from donating blood, tissues and semen. They should also stay under observation for 21 days after their exposure.^8^ Health workers exposed to monkeypox but asymptomatic should also be allowed to continue working with appropriate personal protective equipment (PPE).^19^

## Vaccination for monkeypox

**FIGURE 4:**
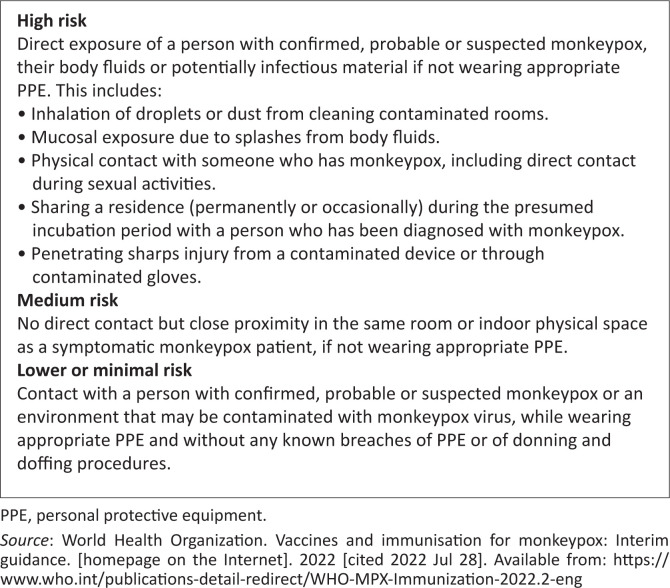
Assessed level of risk of the possible types of exposure.

Mass vaccination is not recommended for people with potential exposure to monkeypox.^22^ All decisions regarding the immunisation of monkeypox should be based on a joint assessment of the risks and benefits of the vaccines by a healthcare provider and a prospective vaccinee.^2,22^ Figure 4 demonstrates the assessed risk level of the possible exposure types. Postexposure prophylaxis (PEP) can be used to prevent the onset of disease in people with a high to medium exposure to monkeypox,^22^ and the general recommendation is to be given within 4 days of exposure to the virus. It can also be given up to 14 days in the absence of symptoms, particularly for individuals at high risk of experiencing ongoing exposure.^2,6,22^

Pre-exposure prophylaxis is recommended for healthcare workers and individuals at high risk of potential exposure to the virus.^22^ This can also be offered to individuals performing diagnostic tests for monkeypox.

Although the effectiveness of the smallpox vaccine has been known to be 85%, following the smallpox eradication in 1980, routine vaccinations against the disease were no longer advised.^2,22^ The original first-generation smallpox vaccine has been removed from the market due to the development of safer vaccines. Newer second- and third-generation vaccines developed for smallpox have been approved for use against monkeypox.^22^ For patients with immunosuppression, these smallpox vaccines have been contraindicated and can be replaced with vaccinia immune globulin.^18^

## Prevention of monkeypox

**FIGURE 5:**
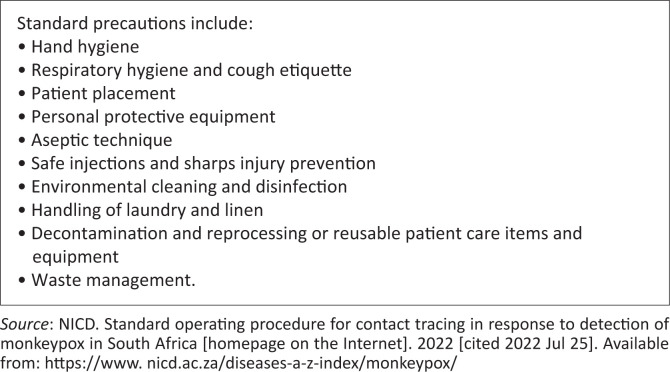
Infection prevention and control standard operating procedure for healthcare management of monkeypox patients.

Infection control and prevention (IPC) are essential steps to prevent the spread of monkeypox. These include regular hand hygiene, the use of disinfectants and the proper disposal of needles and other medical equipment.^23^ Besides these, other precautions such as gloves and practising adequate respiratory hygiene are essential to prevent the spread of monkeypox infections.^6,23^ Figure 5 demonstrated the standard precautions to be followed by all healthcare workers when controlling the spread of monkeypox. However, there is a need for additional risk assessment to evaluate the effectiveness of these measures.

## Laboratory diagnosis of monkeypox

A case that meets the diagnostic criteria of either a probable or suspected case can be confirmed by the presence of viral DNA. This can be carried out through polymerase chain reaction or sequencing.^24^ In addition, a positive result for an orthopoxvirus serological test can be obtained if the patient has not been vaccinated against smallpox or exposed to other viral infections.^18,24^ The ideal samples for monkeypox come from the skin lesions (vesicle or pustule skin or fluid, scab or dry crust).^24^ Other optional specimens that can be sent case-by-case in consultation with National Institute for Communicable Diseases (NICD) are throat swabs, rectal and/or genital swabs (if lesions present), semen, plasma or serum.

## Management of monkeypox

Most people infected with the monkeypox virus do not require specific treatment. Clinical management of cases aims to reduce morbidity and minimise transmission of disease. The disease is usually self-limited and can last up to 4 weeks.^16^ However, public health measures can be used to control the spread of monkeypox. These include early diagnosis, isolation and contact tracing.^6,16^ Follow the IPC steps to minimise the spread of the disease. If a person has been diagnosed with monkeypox and has been established as a confirmed case, they should be isolated until the skin lesions have turned into crusts and the scabs have disappeared.^8^

When caring for an infected person, the caregiver must avoid close contact, and the patient’s lesions should be covered with a bandage or clothing. The following care is suggested for the skin and mucous membrane lesions of monkeypox:^6,16,19^

- The affected areas of the skin need to be cleaned with warm and soapy water and a povidone-iodine solution.
- Vitamin A supplementation is recommended to prevent corneal scarring and visual impairment.
- Use special protective glasses or pads to prevent the eyes from getting damaged.
- Performing a mouth wash with clean, warm salted water is recommended for mouth lesions.
- To minimise the pain caused by mouth ulcers, use an oral analgesic medication and encourage adequate fluid intake.
- Administer oral or topical antibiotics as needed to treat secondary bacterial infection.

Both smallpox and monkeypox viruses are genetically similar. The vaccines and antiviral drugs developed for smallpox are effective against monkeypox. New generation smallpox vaccines have been approved against monkeypox as pre- and post-exposure prophylaxis.^22^ In January 2022, the European Medicines Agency approved using tecovirimat and brincidofovir (antiviral agents) for treating monkeypox.^2,18^ However, the availability of tecovirimat is currently limited to a specific country.

## Complications of monkeypox

Complications of the disease can include various conditions such as pneumonitis, encephalitis and meningitis.^7,16^ One of monkeypox’s recognised and common long-term complications is the formation of pitted scar tissue.^16^ Although the case fatality rate for unvaccinated individuals can range from around 0% – 11%, immunocompromised patients are more prone to experiencing more severe complications.^22^

## Prognosis of monkeypox

The symptoms of the disease are usually mild and self-limiting. However, it can be severe among children, pregnant women and individuals with immune suppression.^16^ Having a residual immunity from the previous smallpox vaccination significantly reduces the severity and frequency of the clinical signs and symptoms of the disease.^22^

## Conclusion

In conclusion, the monkeypox virus has raised many important questions regarding its potential for rapid spread, especially after recent experiences with the ongoing coronavirus disease 2019 (COVID-19) pandemic. For instance, concerns may arise among lay community members that the monkeypox virus can develop new properties and mutate into a more contagious disease. Although there is currently no evidence that transmission by individuals can sustain zoonotic infections in humans, the continued emergence of these pathogens highlights the interconnectedness of animals and humans. The importance of implementing a ‘One Human–Environmental–Animal Health’ approach to controlling zoonotic infections must be acknowledged by policymakers.

As monkeypox is a contagious disease, frontline primary healthcare workers should be on the lookout for additional symptoms and signs suggestive of the monkeypox case definition when dealing with patients presenting with an unexplained acute skin rash. Currently, there is no definitive treatment, but new-generation smallpox vaccines have been demonstrated to be very effective against monkeypox disease. As a part of the management strategy, primary care and public health professionals are expected to increase their efforts to be vigilant and contain any potential outbreaks.
